# Matrix-bound nanovesicle-associated IL-33 supports functional recovery after skeletal muscle injury by initiating a pro-regenerative macrophage phenotypic transition

**DOI:** 10.1038/s41536-024-00346-2

**Published:** 2024-01-27

**Authors:** J. G. Bartolacci, M. N. Behun, J. P. Warunek, T. Li, A. Sahu, G. K. Dwyer, A. Lucas, J. Rong, F. Ambrosio, H. R. Turnquist, S. F. Badylak

**Affiliations:** 1 Department of Bioengineering, University of Pittsburgh, Pittsburgh, PA USA; 2 Departments of Surgery and Immunology, University of Pittsburgh Medical Center, Pittsburgh, PA USA; 3 Department of Physical Medicine and Rehabilitation Sciences, University of Pittsburgh, Pittsburgh, PA USA; 4 McGowan Institute for Regenerative Medicine, Pittsburgh, USA

**Keywords:** Regenerative medicine, Tissue engineering

## Abstract

Injuries to skeletal muscle are among the most common injuries in civilian and military populations, accounting for nearly 60% of extremity injuries. The standard of care for severe extremity injury has been focused upon limb salvage procedures and the utilization of tissue grafts or orthotics in conjunction with rehabilitation to avoid amputation. Nonetheless, many patients have persistent strength and functional deficits that permanently impact their quality of life. Preclinical and clinical studies have shown that partial restoration of functional skeletal muscle tissue following injury can be achieved by the implantation of a biologic scaffold composed of extracellular matrix (ECM). These favorable outcomes are mediated, at least in part, through local immunomodulation. The mechanisms underlying this immunomodulatory effect, however, are poorly understood. The present study investigates a potential mechanistic driver of the immunomodulatory effects; specifically, the effect of selected ECM components upon inflammation resolution and repair. Results show that the host response to skeletal muscle injury is profoundly altered and functional recovery decreased in *il33*^−/−^ mice compared to age- and sex-matched wildtype counterparts by 14 days post-injury. Results also show that IL-33, contained within matrix-bound nanovesicles (MBV), supports skeletal muscle regeneration by regulating local macrophage activation toward a pro-remodeling phenotype via canonical and non-canonical pathways to improve functional recovery from injury compared to untreated *il33*^−/−^ counterparts. Taken together, these data suggest that MBV and their associated IL-33 cargo represent a novel homeostatic signaling mechanism that contributes to skeletal muscle repair.

## Introduction

Skeletal muscle tissue has substantial regenerative ability following injury, an inherent property that relies on the coordinated recruitment, activation, and differentiation of resident stem/progenitor cells^1–3^. Spatial and temporal control of this stem/progenitor cell activity is orchestrated by and dependent upon specific immune cell types, especially macrophages^4,5^. The injury response begins with a robust infiltration of neutrophils within the first hours of injury, rapidly followed by the accumulation of pro-inflammatory, ‘M1-like’ macrophages^5–7^. These pro-inflammatory macrophages promote the expansion of the myoblast pool via the cytokine and chemokine effects of their secretome upon resident stem/progenitor cells^5,8^. These pro-inflammatory macrophages transition to a pro-regenerative, ‘M2-like’ macrophage phenotype between 4- and 14-days post-injury^5^. The secretome of the M2-like macrophages promotes myoblast differentiation into myotubes, in conjunction with the appearance of adaptive immune cells^5,8–10^. The temporal switch from M1-like to M2-like macrophages is required for functional muscle repair^7,11^, and a failure to transition has been associated with delayed healing^9^. Phagocytosis of apoptotic neutrophils may contribute to this macrophage phenotype transition, but a definitive mechanism for this process has not been convincingly established^12^.

The macrophage phenotype transition is notably absent after large muscle injuries where a pro-inflammatory macrophage response is sustained and associated with minimal tissue regeneration and downstream functional impairment^8,11,13^. The surgical implantation of bioscaffolds composed of acellular mammalian extracellular matrix (ECM) has been shown to partially restore both structure and function in animal models of muscle injury^7,11,14,15^ and in humans^16^. The favorable outcomes are believed to be mediated, at least in part, by the promotion of an M-2 like macrophage phenotype that promotes constructive remodeling^8,14,16,17^. Matrix-bound nanovesicles (MBV) that reside within the ECM have been identified as a major contributor to the immunomodulatory effects of ECM^15,18^. Characterization of MBV cargo shows significant amounts of interleukin-33 (IL-33)^19^, a unique protein that is typically sequestered in the nucleus of stromal cells, including fibro/adipogenic progenitor (FAP) cells of muscle tissue^20^. Shortly after tissue injury, the release of IL-33 from damaged or stressed cells targets immune cells expressing the IL-33 receptor, Stimulation-2 (ST2), to induce pleiotropic local and systemic effects^21–23^. IL-33 has been identified to play an important role in the host response to skeletal muscle injury. Specifically, IL-33 promotes accumulation of FoxP3^+^ T_REG_ cells that secrete amphiregulin, a cytokine that supports muscle satellite cells^20,24^. IL-33 contained within the lumen of MBV however can function independent of the ST2 receptor and has separate and distinctive effects upon cell behavior, including alternative macrophage phenotype activation^19^.

The objective of the present study was to determine the in vitro and in vivo effects of MBV-associated IL-33 on macrophage phenotype and associated muscle regeneration following muscle injury in a rodent model. Results show that MBV and their associated IL-33 cargo reduce M1-like and promote M2-like gene transcription in vitro, and promote myogenesis in vivo. In vivo, genomic deletion of IL-33 resulted in a sustained pro-inflammatory response and reduced functional recovery following skeletal muscle injury. The defect in functional recovery could be partially ameliorated through the exogenous provision of MBV containing IL-33. Taken together, these data show that MBV-mediated delivery of IL-33 promotes a macrophage phenotype transition that contributes to functional skeletal muscle repair.

## Results

### Genetic deletion of IL-33 impairs functional recovery and promotes a pro-inflammatory response to skeletal muscle injury

**Fig. 1:**
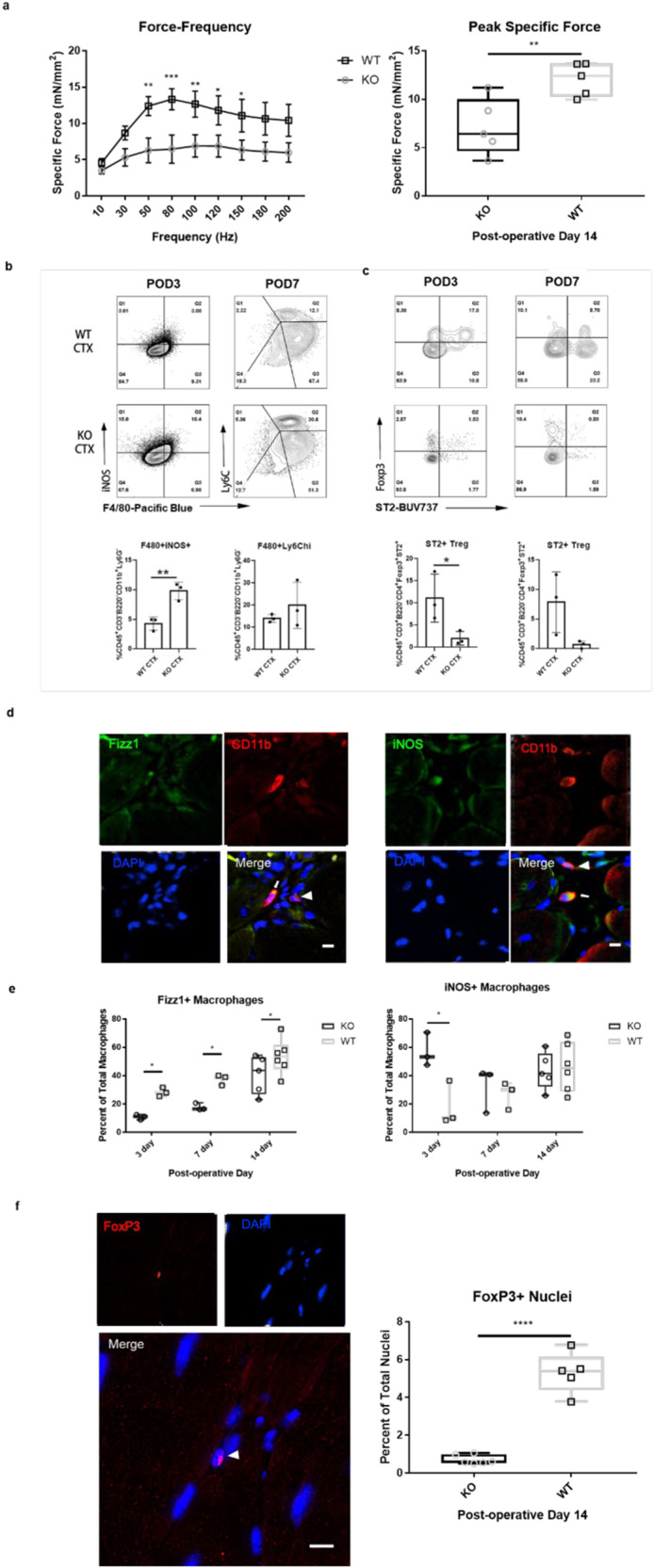
Genetic deletion of IL-33 impairs functional recovery from skeletal muscle injury and promotes a pro-inflammatory immune response. Cardiotoxin muscle injuries were performed on 8 week old B6-*il33*^*+/+*^*arg1*^GFP^ (WT) or B6-*il33*^*−/−*^*arg1*^GFP^ (KO) mice. Functional analysis was performed on POD14 male mice. Flow cytometric analysis or in situ immunolabeling was performed on male or female mice sacrificed at POD3, POD7, and POD14. **a** In situ contractile testing comparing *il33*^−/−^ vs. *il33*+*/+* mice reveals significantly different force-frequency responses (*p* < 0.05, data shown as mean ± SEM) and reduced peak specific force in il33^−/−^ mice compared to wildtype counterparts (*p* < 0.01). **b** Representative dot plots and frequency for inflammatory macrophages in the CD45^+^CD3^-^B220^-^CD11b^+^Ly6G^-^ gate (data shown as mean ± SEM). **c** Dot plots and frequency for ST2^+^ Treg in the CD45^+^CD3^+^B220^-^CD4^+^ gate (data shown as mean ± SEM). **d** Representative in situ immunolabeling images showing CD11b+ macrophages (arrowheads), CD11b+Fizz1+ (arrows, left panel), or CD11b+iNOS+ (arrows, right panel). **e** Quantification of immunolabeling shows significantly fewer Fizz1+ macrophages in il33^−/−^ mice at all timepoints (*p* < 0.05), and increased iNOS+ macrophages at POD3 (*p* < 0.05). **f** Representative in situ immunolabeling image shows nuclear FoxP3 staining (left panels). Quantification of FoxP3+ T_REG_ immunolabeling shows reduced FoxP3+ T_REG_ accumulation in il33^−/−^ animals compared to wildtype counterparts. (Scale bars = 5 µm, *N* = 5 biological replicates, *n* ≥ 3 technical replicates. Results shown as Min-Max unless otherwise specified, *p*-values were calculated using repeated measures ANOVA (force-frequency), two-tail t-test (peak specific force and FoxP3 immunolabeling), one-way ANOVA (flow cytometry), or multiple t-tests with two stage step-up method of Benjamini, Krieger and Yekutieli false discovery rate p-value correction (immunolabeling), *p < 0.05, **p < 0.01, ****p < 0.0001).

Cardiotoxin (CTX) injury of the tibialis anterior (TA) muscle compartment in mice between 6-8 weeks of age was used to induce a reproducible, regenerative muscle injury. Repeated measures two-way ANOVA was performed to determine the effect of genotype on specific force production at post-operative day (POD)14 following CTX injury. Results show that there was no interaction effect, and that genotype was a significant effect at multiple stimulation frequencies (25% of total variation, *p* < 0.0001; Fig. 1a left panel). Multiple comparisons showed significantly higher force production by wild type animals at stimulation frequencies between 50–150 Hz compared to *il33*^*−/−*^ littermates (*p* < 0.01, *p* < 0.001, *p* < 0.01, *p* < 0.05, and *p* < 0.05, respectively; Fig. 1a). Further, peak specific force, or the maximum specific force produced by a biological replicate at any stimulation frequency, was lower in *il33*^−/−^ animals compared to wildtype counterparts (33% reduction, *p* < 0.01; Fig. 1a, right panel). This was also reflected in decreased numbers of MyoD^+^ activated satellite cells (Supplementary Fig. 1). Taken together, these data show that depletion of IL-33 results in an inferior regenerative response compared to animals with a normal amount of IL-33. Importantly, these differences did not arise from baseline differences in function (Supplementary Fig. 2) or local immune populations between *il33*^−/−^ and wildtype, as assessment of macrophage and Treg populations of untreated skeletal muscle did not show any significant defects in these populations (Supplementary Figs. 3, 4).

The injured skeletal muscle of CTX-treated animals was harvested at post-operative day (POD)3, POD7, POD14, and POD28, and the mononuclear immune cells present in injured muscle at these timepoints were assessed by flow cytometry. Results show that the CD45^+^CD3^−^B220^−^CD11b^+^Ly6G^−^F480^+^iNOS^+^ M1-like macrophage population was nearly 2x greater in muscle tissue from *il33*^−/−^ mice than *il33*^*+/+*^ littermates at POD3 (10% vs. 5%, respectively, *p* ≤ 0.01; Fig. 1b). By POD7, the M1-like macrophage infiltrate trended higher in *il33*^−/−^ mice (~20%) compared to *il33*^*+/+*^ mice (~15%), though these differences were not statistically significant at this time point. CD45^+^CD3^+^B220^−^CD4^+^Ly6G^-^FoxP3^+^ST2^−^ T_REG_ cells were also significantly less abundant in *il33*^−/−^ mice than in *il33*^*+/+*^ littermates at POD3 (~2.5% vs. ~11%, *p* < 0.05; Fig. 1c). Potential evidence for sustained inflammation and injury at day 28 was observed in *il33*^−/−^ mice as high numbers of Treg (Supplementary Fig. 5a) and Macrophages (Supplementary Fig. 5b) persisted relative to WT.

Flow cytometry results were corroborated by immunolabeling, which showed that the phenotypic composition of the macrophage populations was strikingly different between strains. CD11b^+^Fizz1^+^ M2-like macrophages represented a greater percentage of macrophages at all time points in *il33*^*+/+*^ mice compared to their *il33*^−/−^ counterparts (*p* < 0.05 for POD3, POD7, and POD14; Fig. 1d,e). Further, the macrophage infiltrate in *il33*^−/−^ mice consisted of CD11b^+^iNOS^+^ M1-like macrophages to a greater extent than *il33*^*+/+*^ mice at POD3 (*p* < 0.05, Fig. 1d, e). Immunolabeling for FoxP3^+^ T_REG_ cells show significantly reduced accumulation in *il33*^*−/−*^ compared to wild-type animals (*p* < 0.0001, Fig. 1f), in accordance with previous reports^20^. These data establish that a functional defect in recovery after skeletal muscle injury is associated with an early augmentation in INOS^+^ macrophages and a deficit of Fizz1^+^ macrophages and ST2^+^ Treg.

### Exogenous provision of MBV-associated IL-33 partially restores macrophage phenotype distribution and function

**Fig. 2:**
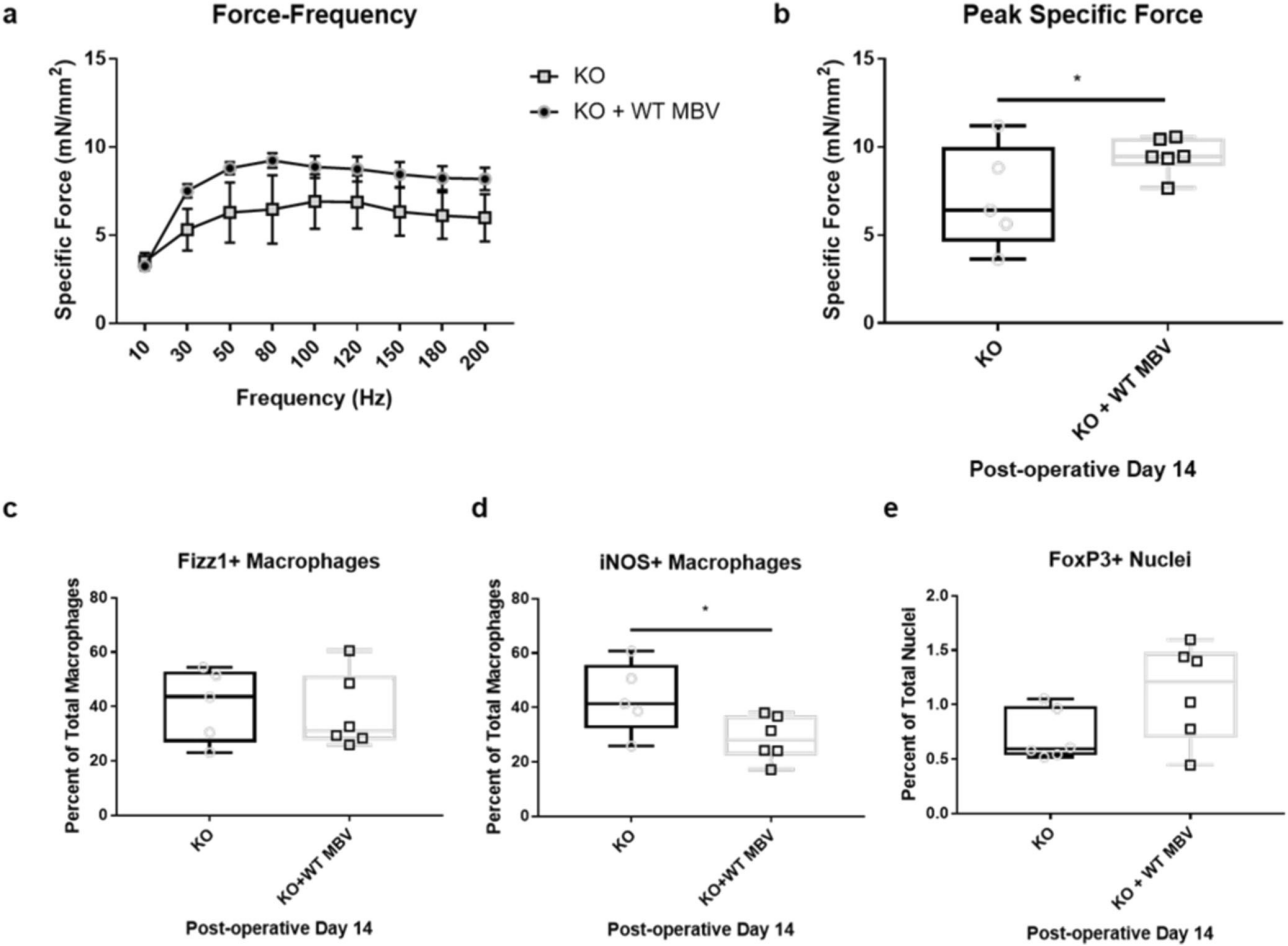
Exogenous provision of IL-33^+^ MBV partially restores function and macrophage phenotype response to skeletal muscle injury. **a**, **b** In situ contractile testing performed at POD14 on untreated *il33*^−/−^ mice (KO) or *il33*^−/−^ mice to which IL-33 + MBV were delivered (KO + WT MBV) intramuscularly. Peak specific force was significantly greater in treated animals compared to untreated counterparts (*p* < 0.05). (**c-d**) In situ immunolabeling of macrophages revealed significantly fewer iNOS positive macrophages in IL-33 + MBV-treated mice compared to no treatment (*p* < 0.05). **e** Quantification of FoxP3 immunolabeling shows no significant difference in Treg cell accumulation as a result of MBV treatment. (*N* = 5 biological replicates for KO mice, *N* = 6 biological replicates for KO mice treated with IL-33 + MBV, *n* ≥ 3 technical replicates. Data are presented as mean ± min/max (peak force) or mean + SEM (Force-Frequency), independent variables were tested for significance using two-way ANOVA (Force-Frequency), two tail t-tests, * denotes *p* < 0.05).

To determine if MBV and their associated cargo play a role in the modulation of macrophage phenotype in vivo, IL-33^+^ MBV were injected into the tibialis anterior muscle of cardiotoxin-injured *il33*^*−/−*^ mice on POD2. Results of functional testing and immunolabeling show that injection of 5 × 10^10^ MBV directly into the injured TA muscle of *il33*^*−/−*^ mice resulted in a peak specific force that was increased compared to their untreated *il33*^*−/−*^ counterparts (*p* ≤ 0.05) by POD14. Upon completion of the functional testing regimen, TA muscles used for in situ contractile testing were harvested and used for immunolabeling of macrophage infiltrate and FoxP3^+^ T_REG_ cells. A two-way ANOVA was performed to examine the effect of IL-33^+^ MBV treatment on specific force generation at different stimulation frequencies. Results show a significant treatment effect (10.8% of total variation, *p* = 0.0002), as well as a significant frequency effect (20.5% total variance, *p* = 0.01; Fig. 2a). Further, IL-33^+^ MBV resulted in increased peak specific force generation (33% increase, *p* < 0.05; Fig. 2b) compared to the untreated counterparts from Fig. 1a. Immunolabeling data showed fewer CD11b^+^iNOS^+^ M1-like macrophages in IL-33^+^ MBV-treated animals compared to untreated *il33*^*−/−*^ mice by POD14 (*p* < 0.05, Fig. 2d), however, there was no difference in CD11b^+^Fizz1^+^ macrophages (Fig. 2c) or FoxP3^+^ T_REG_ cell accumulation (Fig. 2e) as a function of treatment in response to IL-33^+^ MBV treatment at this time point. These data establish that delivery of MBV was able to improve functional repair after muscle injury and the increase in function was associated with a significant phenotypic change in local macrophage population.

### MBV-associated IL-33 signals independently of the ST2 receptor and promotes myogenesis through a macrophage-mediated mechanism

**Fig. 3:**
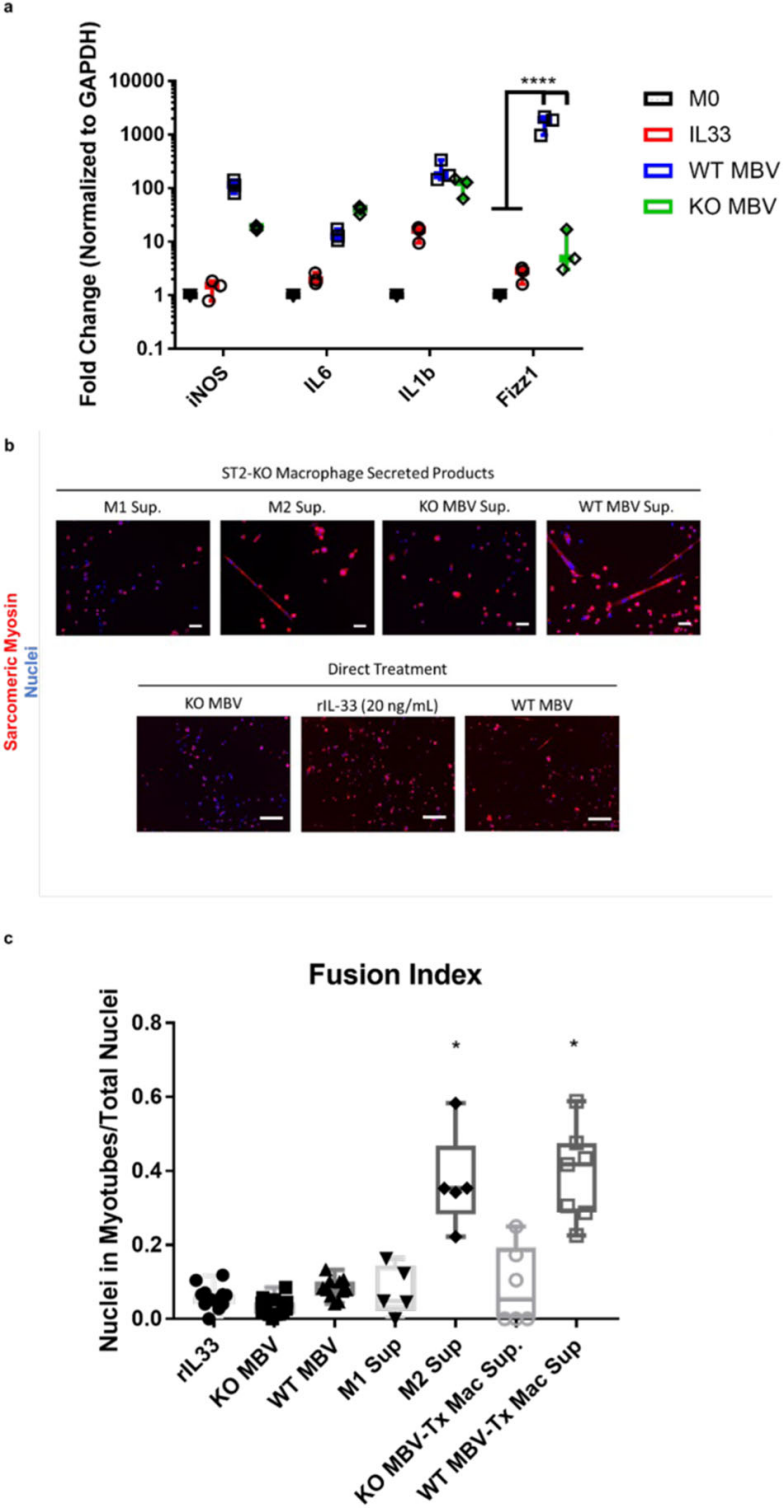
MBV-associated IL-33 promotes M2-like gene expression and macrophage-mediated differentiation of primary muscle stem cells. **a** Quantitative polymerase chain reaction assay measuring gene expression changes of nos2 (iNOS), il6 (IL6), il1b (IL1b), and fizz1 (Fizz1), **b**, **c** MuSC were treated for 4d with the secreted products of st2^−/−^ macrophages exposed to: IFNg+LPS (M1 sup), IL-4 (M2 sup), IL-33- MBV (KO MBV Tx Mac Sup), or IL-33 + MBV (WT MBV Tx Mac Sup), or 1 × 109 IL-33^+^ MBV (WT MBV), 1 × 109 IL-33^−^ MBV (KO MBV), or 20 ng/ml rIL-33 (rIL33). Fixed cells were immunolabeled for myosin heavy chain to identify mature myotubes. Myotube fusion index (# of nuclei in myotubes with ≥ 3 nuclei / Total # of Nuclei) differences were assessed using one-way ANOVA. Gene expression changes were assessed using two-way ANOVA. Significant differences were interrogated using post-hoc testing. (Scale bars = 25 µm, *N* = 3 replicates, * denotes *p* < 0.05, **** denotes *p* < 0.0001).

To investigate the processes being regulated by MBV and their associated cargo, qPCR was performed on RNA derived from bone marrow-derived *st2*^−/−^ macrophages that had been exposed to one of the following treatments: 1 × 10^9^ IL-33^+^ MBV, 1 × 10^9^ IL-33^−^ MBV, 20 ng/mL rIL-33, or media control. Results show that MBV-associated IL-33 promoted a unique pattern of gene expression compared to either rIL-33 or IL-33^−^MBV, and promoted significant upregulation of Fizz1 expression compared to other treatments (*p* < 0.0001; Fig. 3A). Studies have shown that MBV recapitulate the effects of whole ECM on macrophage phenotype in vitro, and that MBV-treated macrophages promote myoblast differentiation^19,25^; however, the direct and indirect effects of soluble or MBV-associated IL-33 on primary MuSC fate has not been evaluated. Consistent with previous studies^8,13^, secreted products of *st2*^−/−^ macrophages exposed to IL-4 and IL-33^+^ MBV resulted in greater indices of MuSC differentiation than MuSC treated with the secretomes of IFNγ + LPS or IL-33^−^ MBV-treated macrophages (*p* < 0.05; Fig. 3b, c). Direct MuSC treatment with MBV, with or without IL-33, or recombinant IL-33 did not promote differentiation (Fig. 3b, c). These data show that IL-33 contained with MBV can promote expression of M2-associated marker Fizz1 in an ST2-independent manner. Further, MBV promote myogenesis in vitro through a macrophage-mediated mechanism.

### While MBV promote macrophage differentiation at the injury site independently of the ST2 receptor, MBV restoration of function requires ST2

**Fig. 4:**
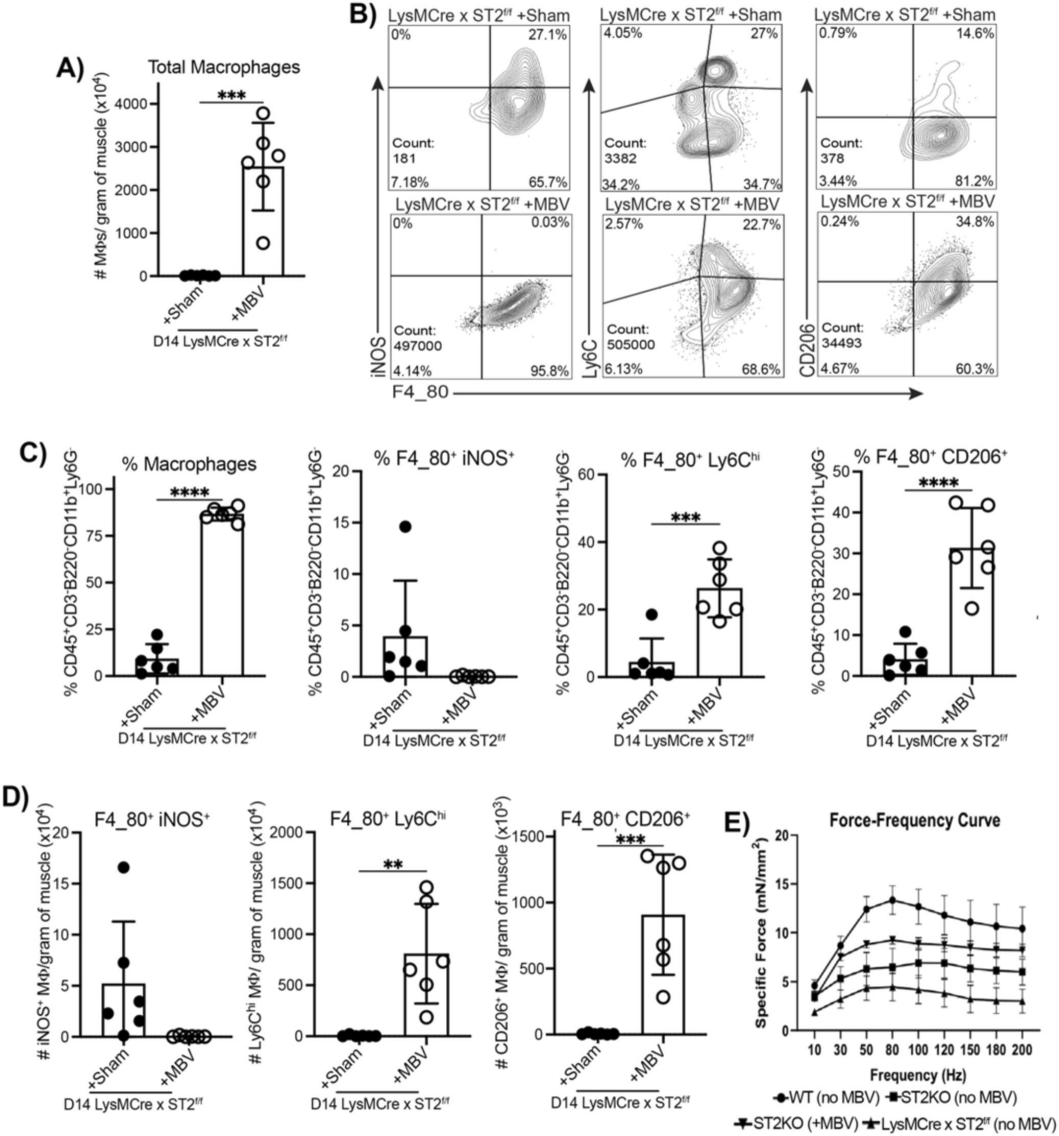
MBV administration increases CD206+ macrophages independently of macrophage-specific ST2 signaling. **A** Absolute macrophages per gram of muscle 14 days post LysMcre-ST2^f/f^ injury with cardiotoxin and co-administration with vehicle or MBV. **B** Representative flow plots of F4_80^+^ macrophages expressing iNOS, Ly6C and CD206 14 days post LysMcre-ST2^f/f^ injury with cardiotoxin and co-administration with vehicle or MBVs. **C** Frequency quantification of F4_80^+^ macrophages expressing iNOS, Ly6C or CD206 out of CD45 live cells. **D** Absolute macrophages per gram of muscle expressing iNOS, Ly6C or CD206. **E** Absence of macrophage ST2 reduces the beneficial impact of MBV treatment on force-producing capacity of mice. Force-frequency curves for TA stimulation in mice from four different experimental groups. (*N* = 5-6/group, two-way ANOVA with repeated measures, * denotes *p* < 0.05, ** denotes *p* < 0.005, *** denotes *p* < 0.001). *N* = 6 per group from 2 independent experiments. Error bars are SD. Student’s t-test.

We next assessed how the absence of ST2 on macrophages impacted MBV-mediated modulation of macrophage phenotype in vivo and restoration of muscle function. IL-33^+^ MBV were injected into the tibialis anterior muscle of cardiotoxin-injured *il33*^−/−^ (KO) mice or mice whose macrophages are deficient for ST2 (LysM-Cre × *St2*^fl/fl^) mice on POD2 and compared to injured *il33*^+/+^ (WT) or *il33*^−/−^ mice alone. Assessment of immune populations again revealed that MBV-mediated ST2-independent reduction of iNOS^+^ macrophages, while increasing CD206^+^ macrophages at the site of injury at POD14 (Fig. 4). However, functional testing suggested that IL-33-mediated signaling to macrophages was also important for the restoration of muscle function (Fig. 4E). Specifically, while force production was augmented by MBV in *il33*^−/−^ mice, LysM-Cre × *St2*^fl/fl^ treated with MBV were unchanged functionally (Fig. 4E). In total, these data establish that delivered MBV can modulate local macrophage differentiation, but both canonical and non-canonical activities are needed to fully enable macrophage function in the restoration of function after muscle injury.

## Discussion

Results of the present study show that MBV-delivered IL-33 promotes a macrophage secretome independent of ST2 that initiates the activation and differentiation of skeletal muscle satellite cells in vitro. Furthermore, the local administration of MBV-associated IL-33 at the sites of acute, severe muscle injury is associated with increased functional recovery following injury and the generation of CD206^+^ macrophages and suppression of iNOS^+^ pro-inflammatory macrophages at the injury site. The present study identifies the delivery of IL-33 via MBV as a potentially practical, yet powerful method to improve outcomes after muscle injury.

Following the loss of cell integrity, fully active nuclear IL-33 is released to the extracellular space where it can be cleaved by proteases to produce a more active form. When IL-33 complexes with the ST2 receptor on infiltrating immune cells it produces pleiotropic outcomes that continue to be elucidated^21^. It is understood that the IL-33/ST2 axis is essential for the accumulation of T_REG_ cells in skeletal muscle healing^20^ and the programming of macrophage metabolism which supports differentiation into reparative subsets^26^. However, the current study and recent reports show that MBV-associated IL-33 may also influence macrophage phenotype through poorly characterized ST2-independent mechanisms^19,27^ One mechanism may involve the recent demonstration that IL-33 delivered in MBV can travel to the nucleus and instruct an anti-inflammatory phenotype in both wildtype and *St2*^−/−^ macrophages by upregulating M2-like and downregulating M1-like gene transcription^27^. While the role of ST2^+^ T_REG_ have not been assessed in the present study, our in vitro and in vivo examinations utilizing ST2-deficient macrophages clearly show the functionality of an ST2-independent IL-33 pathway that modulates the differentiation and the secretome of macrophages. Results of the present study also show that MBV-associated IL-33 reduces the pro-inflammatory macrophage response to skeletal muscle injury in vivo and partially restored functional recovery from skeletal muscle injury. Thus, multiple IL-33-mediated, ST2-dependent and independent mechanisms appear to be of potential importance for effective functional skeletal muscle repair

The direct treatment of MuSC with MBV or rIL-33 had no effect on the differentiation of these cells. Yet, the secreted products of ST2-deficient macrophages treated with IL-33^+^ MBV promoted significantly greater MuSC differentiation than the secretome of IL-33^-^ MBV or rIL-33-treated macrophages. These data indicate that IL-33, delivered via MBV, functions through gene regulation^27^ or by another yet-to-be-identified mechanism that can direct macrophage-mediated myogenic differentiation of MuSC. Previous reports have shown that MBV and their IL-33 cargo promote a pro-healing, M2-like macrophage phenotype, which, together with the results of the present study, suggests that MBV-associated IL-33 may feedback on target cells to promote the resolution of inflammation and promotion of constructive tissue remodeling^19^. Additional studies are needed to evaluate potential compensatory MBV changes resulting from IL-33 deletion; however, the present work provides evidence for a novel paradigm for gene expression regulation when delivered by way of extracellular vesicles.

Although ECM bioscaffolds have repeatedly been shown to promote an M2-like macrophage response in mouse models of muscle injury^8,17,28,29^, the present study offers evidence that a single component of the ECM can modulate macrophage phenotype in vivo, independent of the parent ECM biomaterial. In vivo results show that genomic deletion of IL-33 is primarily associated with an augmented pro-inflammatory state following skeletal muscle injury, especially in the acute post-injury phase. The macrophage response in *il33*^−/−^ deficient mice to injury was comprised predominantly of pro-inflammatory M1-like macrophages, with lesser numbers of pro-healing M2-like macrophages than their *il33*^*+/+*^ littermates. As a consequence of this M2-like:M1-like imbalance, *il33*^−/−^ mice showed a blunted satellite cell activation at POD3, culminating in reduced functional recovery from injury by POD-14. Immunological evidence of unresolved injury persisting at POD28 was found in both ST2- and IL-33 deficient mice (Supplementary Fig. 1). In accordance with previous studies, loss of IL-33 in skeletal muscle tissue resulted in diminished functional recovery from injury by way of reduced ST2-mediated FoxP3^+^ T_REG_ cell accumulation that could be ameliorated through exogenous provision of IL-33^20^. Results of the present study also show a reduced accumulation of FoxP3^+^ T_REG_ cells in *il33*^−/−^ mice following injury. One salient finding of the present study is the observation that IL-33^+^ MBV delivery did not result in profound increases in T_REG_ cell accumulation compared to untreated *il33*^−/−^ counterparts. This finding is distinct from previous observations when IL-33 is delivered systemically^20^, and these results strongly suggest that the macrophage phenotype transition, mediated by MBV-associated IL-33, is a critical contributor to functional skeletal muscle repair. The role of systemic changes as a result of MBV treatment in promoting increases in functional recovery from skeletal muscle injury cannot be ruled out. The contralateral leg could not be used as a comparator control and all comparisons were made with respect to untreated *il33*^−/−^ mice. It should be noted that while the force production of *il33*^*+/+*^ mice equaled that of their uninjured counterparts (Supplementary Fig. 1c), the force production of *il33*^*−/−*^ mice was significantly less. It is therefore possible that IL-33 deletion delays healing, and justify a study that evaluates functional recovery at time points further from the injury date. It is of note that MBV treatment did not fully recapitulate wild-type functional recovery by POD14.

In addition to the current limited assessment of ST2-Deficient Macrophages, future work using cell-type specific deletions of the ST2 receptor is needed to clearly elucidate non-canonical and axis contributions to skeletal muscle repair. Figures 3 and 4 clearly show that IL-33^+^, but not IL-33^-^ MBV, can act on ST2-deficient macrophages to induce Fizz1 (Fig. 3a) and generate a secretome able to promote myogenesis (Fig. 3c). Likewise, IL-33^+^ MBV generated increased local CD206+ macrophages while reducing the presence of iNOS+ macrophages at the injury site in mice with ST2-deficient macrophages (Fig. 4). Yet, these data, combined with past demonstrations that ST2 expression by Treg^30,31^ and macrophages^32^ support repair and immune regulation, suggest complex immunobiology involving IL-33 as a potent regulator of repair responses after muscle injury. Further studies utilizing now available Treg and macrophage specific and inducibile ST2 deletion should provide answers to when and how IL-33 canonical and non-cononical resposnes are important to skeletal muscle repairl.

The nature of an ST2-independent mechanism remains poorly understood. It has previously been shown that IL-33 translocates from the nucleus to the cytoplasm in response to various cellular stimuli^8,33^. Further, it has been shown that overexpression of IL-33 within cells reduces their responsiveness to extracellular inflammatory signals, resulting in reduced translocation of NFκB and reduced expression of inflammatory mediators^9–11^. Therefore, it is plausible that MBV-delivered IL-33 may regulate gene expression in target cells independent of the ST2 receptor. Our recent publication establishes that such a mechanism is active in macrophages in vitro^27^. Expanded studies utilizing single-cell gene expression analysis techniques, such as single-cell RNA sequencing combined with ATACseq, could directly interrogate genes downstream of the ST2 receptor and directly assess the contribution of ST2 and non-ST2-mediated IL-33 upon skeletal muscle healing.

Previous work has shown that the macrophage phenotype is plastic and can transition from M1-like macrophages toward an M2-like macrophage phenotype in vitro upon stimulation with solubilized ECM products^28,30,34^. The present study shows that a single ECM bioscaffold component, IL-33 contained within MBV, can modulate macrophage phenotype in vivo. One limitation to the present study is that it used a limited number of markers to characterize the phenotype of infiltrating macrophages. The use of multiple markers could be employed in future studies to better characterize the functional phenotype of macrophages. Although a concomitant increase in Fizz1 expression in vivo was not found, skeletal muscle macrophages may nonetheless behave as M2-like macrophages. However, the cellular target(s) by which MBV-associated IL-33 promotes increased functional repair is not known, and a complete characterization of all cell types involved in the host response to skeletal muscle injury was beyond the scope of the present work. Further, the optimal method by which MBV are delivered to maximize skeletal muscle healing should be considered as an area for future investigation. Due to technical limitations, the minimum volume into which 10^10^ MBV could be solubilized was 30 μL; a volume that could potentially compress local TA tissue structures and impact healing. Studies to improve the preparation and resuspension of MBV may identify methods to improve functional outcomes. Finally, we utilized CTX injury, which is a common experimental method used to gain an understanding of the immune response to muscle injury and testing of potential therapeutics^35,36^ Yet, the findings in CTX studies may not be broadly applicable to volumetric muscle loss (VML) that impacts people clinically. While we expect that similar MBV-mediated mechanisms would be observed, given the positive impact ECM containing MBV has displayed in experimental^36^ and clinical VML resolution and repair^16^, this will need to be examined in future studies where MBV and IL-33 ability to support robust muscle repair after the loss of all muscle components and local immune populations is defined.

The role of T_REG_ cells is likely synergistic with the effects of MBV-associated IL-33. However, the present study showed that MBV initiate effects at early time points following injury and T_REG_ cells are present at low numbers until ~7–14 days. Therefore, it is plausible that macrophages initiate the tissue remodeling process. MBV were delivered two days after injury because previous studies showed a robust macrophage infiltration at this time and these M1-like, pro-inflammatory macrophages would have had an opportunity to promote the expansion of the MuSC pool^4,5,8,17,31^. The dose of 5 × 10^10^ MBV/muscle was based on the observation that 1 × 109 MBV/mL was sufficient to activate 2 × 10^6^ macrophages in vitro, but optimization of dose and dose regimen remains to be established. Previous work showed that 100 mg of ECM lyophilized yields 10^11^-10^12^ MBV^37^. Typically, 2 × 10^6^ macrophages can be activated in vitro with far less than 100 mg of ECM, which suggests that 10^10^ MBV may provide an excess of particles^19,25,28^. Preferred dose delivery methods have yet to be identified. Repeated exposure of skeletal muscle injury sites to ensure local delivery of MBV could result in the reactivation of the inflammatory innate immune response and reduce the efficacy of IL-33^+^ MBV.

The specific signaling molecules released by macrophages that promote expansion or differentiation of the muscle stem/progenitor cell pools are also unknown. Further, the present study was not designed to assess interactions between innate and adaptive immune cell types, which are likely important for maximum muscle recovery following injury. The importance of ST2-mediated signaling to support Treg-mediated phenotype transition^32^ and sustain metabolic function in macrophages enabling M2 polarization has been previously shown^26^. It will be important in future studies to employ mouse strains with targeted or inducible deletions of the ST2 receptor on T_REG_ cells and macrophages deficient to more precisely define the roles these cells play in repair responses after muscle injury.

In summary, the findings of the present study show a critical role for MBV-sequestered IL-33 after skeletal muscle injury and subsequent healing as IL-33 contained within MBV is required for a macrophage phenotype transition that supports skeletal muscle repair. Our data also suggest that MBV-bound IL-33 can trigger ST2-independent and canonical signaling in macrophages that is needed for full functional resolution after muscle injury. Changes in macrophage phenotype were predictive of functional tissue remodeling outcomes at POD14 and the healing deficiencies noted in *il33*^*−/−*^ mice could be rescued by the provision of IL-33^+^ MBV. Taken together, these data suggest that MBV and their IL-33 cargo are required for ECM-mediated immunomodulation and functional tissue remodeling.

## Methods

### Ethics declaration on animal use

*C57BL/6 (B6) il33*^−/−^ mice were obtained from S. Nakae (University of Tokyo, Tokyo, Japan)^38^. B6-arg^yfp^-*il33*^−/−^ mice were generated by 6 times backcrossing of B6.129S4-^*arg1tm1lky*^/J (B6-arg1^yfp^; The Jackson Laboratory) mice on to the B6 *il33*^−/−^ background at the University of Pittsburgh. *St2*^−/−^ mice were originally generated on a BALB/c background^39^ and then backcrossed to a B6 background before use. All mice were raised in accordance with and with the express approval from the University of Pittsburgh Institutional Animal Care and Use Committee. The B6 *St2*^fl/fl^ mice were provided by Giorgio Trinchieri (National Cancer Institute, Bethesda, Maryland) and crossed to B6.129P2-Lyz2tm1(cre)Ifo/J (The Jackson Laboratory) to generate LysM^Cre^ × *St2*^fl/fl^ mice. Colony maintenance, including bedding and cage changes, was provided by the University of Pittsburgh Division of Laboratory Animals in Research on a weekly basis. Litters were weaned at 21 days of age and genotyping of all breeding pairs was performed to maintain the integrity of the strains. Animals were used as experimental subjects or as a source of tissues and cells. Male and female animals ≥ 8 weeks of age were used to generate MBV, whereas male animals ≥ 8 weeks of age underwent surgical manipulation. Functional testing was performed using male animals. Pain management was achieved using buprenorphine hydrochloride twice daily for 3 days and infection prevention was maintained for 3 days using Baytril. Animal weights were not recorded for the duration of the study as it was assumed that any weight change that occurred over a two-week span would be negligible. Animal weights were recorded at the time of euthanasia for animals undergoing functional testing. Euthanasia was performed in accordance with guidelines using 20–30% v/v inhaled CO_2_ for 8–10 min until signs of life were no longer detectable and death was ensured using cervical dislocation.

### Cardiotoxin injury model

Cardiotoxin muscle injury model was used as previously described^40^. Briefly, a small incision was made in the skin overlying the tibias of anesthetized mice in the supine position. Blunt dissection of the adjacent subcutaneous region was used to expose the tibialis anterior (TA) muscles and 25 μl of 10 µM cardiotoxin (CTX) (Naja Pallida; Sigma Aldrich, St. Louis, MO) was administered by intramuscular injection to the left and right hindlimbs. The incision was subsequently closed using resorbable sutures and the animals allowed to recover.

### Immune Cell Analysis by Flow Cytometry

Leukocytes within skeletal muscle were isolated by mincing tissue and homogenizing with gentleMACS C tubes (Miltenyi Biotec) in RPMI containing 350 U/mL type IV collagenase (Gibco) and 20 μg/mL DNase I (MilliporeSigma). Tissue was incubated in a shaker at 37 °C at speed 250 RPM for 50 min. Single-cell suspensions were then obtained by passing through a 70-μm cell strainer and purified by density gradient centrifugation using Lympholyte-M (Cedarlane). Cells were incubated with heat-inactivated goat-serum (5%) to block FcR, stained with eFluor 506L/D viability dye (Thermo Fisher) and leukocytes within the damaged muscle tissue were isolated and incubated with heat-inactivated goat-serum (5%) to block FcR, and then labelled with different combinations of fluorochrome-conjugated antibodies (BD Bioscience, Biolegend, eBioscience or MD Biosciences) to distinguish myeloid and T cell populations (CD45(30-F11), CD45.2(104), F4/80(BM8), CD8(53–6.7), CD38(90/CD38), CD4(RM4-5), Foxp3(FJK-16S), Ly6C(AL-21), B220(RA3-6B2), iNOS(CXNF7), CD206(MR5D3), CD3(17A2), CD11b(M1/70), MHCII(2G9), Ly6G(1A8), CD11c(N418), ST2(U29-93,DIH9), EGR2(erongr2), GFP(FM264G)). Data was acquired with either an Aurora-10 (Cytek) or LSRFortessa flow cytometer (BD, Bioscience) and analyzed using FlowJo, Version 10.1 (Tree Star). **Antibodies:** PECF594 Rat Anti-Mouse CD45 (1:400 BD Horizon Cat: 562420; Clone: 30-F11), Pacific Blue Anti-Mouse F4/80 (1:200 BioLegend Cat: 123124; Clone: BM8), BV605 Rat Anti-Mouse CD38 (1:200 BD OptiBuild Cat: 740361; Clone: 90/CD38), BV650 Rat Anti-Mouse CD4 (1:200 BD Horizon Cat: 563232; Clone: GK1.5), PE Rat Anti-Mouse Ly6C (1:200 BD Pharmigen Cat: 560592; Clone: AL-21), PE-Cy5 Rat Anti-Mouse CD45R/B220 (1:200 BD Pharmigen Cat: 553091; Clone: RA3-6B2), Alexa Fluor 700 Rat Anti-Mouse CD3 Molecular Complex (1:200 BD Pharmigen Cat: 561388; Clone: 17A2), APC/Fire 750 Anti-Mouse/Human CD11b (1:200 BioLegend Cat: 101262; Clone: M1/70), BUV395 Anti-Mouse I-A/I-E (1:200 BD OptiBuild Cat: 743876; Clone: 2G9), BUV805 Rat Anti-Mouse Ly6G (1:200 BD OptiBuild Cat: 741994; Clone: 1A8), Brilliant Violet 510 Anti-Mouse CD11c (1:200 BioLegend Cat: 117338; Clone:N418), BUV737 Rat Anti-Mouse IL33R (ST2) (1:100 BD OptiBuild Cat: 749323; Clone: U29-93), PerCP-Cyanine5.5 FoxP3 Monoclonal Antibody (1:200 Thermo Fisher Cat: 45-5773-82; Clone: FJK-16s), PE-Cyanine7 iNOS Monoclonal Antibody (1:400 Thermo Fisher Cat: 25-5920-82; Clone: CXNFT), Alexa Fluor 647 Anti-Mouse CD206 (1:200 BioLegend Cat: 141712; Clone: 068C2), eBioscience Fixable Viability Dye eFluor 506 (1:500 Thermo Fisher Cat: 65-0866-14).

### Skeletal muscle immunolabeling

#### Macrophage immunolabeling

Harvesting of the tibialis anterior was performed at 3, 7, and 14-day post-injury. Each muscle was fixed in 10% neutral-buffered formalin and embedded in paraffin. 5 μm sections were cut and mounted onto glass slides. Slides were deparaffinized using xylene and ethanol gradients (100-70% EtOH). Slides were subjected to antigen retrieval and immunolabeling. Immunofluorescence was performed on serial sections for each subject and timepoint to assess the phenotypes of immune and satellite cell populations. After deparaffinization, the slides were placed in citrate antigen retrieval buffer (10 mM citric acid monohydrate, pH 6.0), microwaved at 100% power for 45 seconds, followed by 15 min at 20% power. The slides were then cooled in copper sulfate solution (10 mM CuSO_4_, 50 mM ammonium acetate, pH 5.0) for 20 min. Sections were then rinsed three times in Tris buffered saline/Tween 20 solution (TBST) and then incubated for 1 hour at room temperature in blocking buffer containing 0.1% Triton-X 100, 0.1% Tween, 2% goat serum, and 1% bovine serum albumin. The blocking buffer was then removed and the sections were incubated overnight at 4 °C in a humidified chamber with 1:200 rabbit-anti-CD11b (ab128797; Abcam, Cambridge, UK), a pan-macrophage marker. Following overnight incubation, each slide was washed in TBST for 3 × 2 min. A 1:200 solution of goat-anti-rabbit horseradish peroxidase (HRP)-conjugated secondary antibody (DAKO, Glostrup, Denmark) in blocking buffer was subsequently applied and microwaved at 40% power for 3 min in a humidified chamber and allowed to cool for 2 min before washing in TBST. After washing, sections were incubated with a 1:200 solution of red fluorescent HRP substrate (OPAL 570; Perkin Elmer, Waltham, MA) in 1x Amplification Diluent (Perkin Elmer) for 10 min and then washed in TBST. To remove anti-CD11b and anti-rabbit antibodies, sections were subjected to a second round of antigen retrieval in citrate antigen retrieval buffer, followed by cooling in copper sulfate solution, and blocked as described above. For each slide, one section was incubated with a 1:200 solution of rabbit-anti-iNOS antibody (PA-0303A; Invitrogen, Carlsbad, CA) in blocking buffer, and one section was incubated with a 1:200 solution of rabbit-anti-RELMα (500-P214; PeproTech, Rocky Hill, NJ). Slides with the primary antibodies were then placed on a raised water bath and microwaved at 40% power for 3 min, followed by 2 min of cooling. Slides were then washed in TBST solution and a 1:200 solution of goat-anti-rabbit HRP-conjugated secondary antibody was placed on the sections. Slides with secondary antibody solutions were then placed in the water bath and microwaved at 40% power for 3 min, followed by 2 min of cooling. After cooling, slides were washed in TBST and a 1:200 solution of green fluorescent HRP substrate in 1x Amplification Diluent (520 Opal, Perkin Elmer) was placed over each section and incubated in a dark humidified chamber for 10 min at room temperature. The sections were then washed in TBST, incubated with 4′,6-diamidino-2-phenylindole (DAPI) nuclear counterstain for 5 min. The sections were washed with TBST and subsequently mounted for imaging by fluorescence microscopy. For each technical replicate, the number of CD11b+ cells was counted and summed to give a total number of CD11b+ cells per biological replicate. For each biological replicate, a minimum of 2000 CD11b+ cells were counted. Co-positive macrophages, or macrophages that expressed CD11b as well as iNOS or Fizz1, the percent of total macrophages was determined by dividing the number of co-positive macrophages by the total number of CD11b+ cells.

#### FoxP3 staining

Paraffin embedded sections were cut and deparaffinized as described above. Antigen retrieval was performed for 20 min at 95–100 °C in citrate antigen retrieval buffer, background fluorescence was quenched using copper sulfate solution and the sections were incubated for 1 h in blocking buffer. After blocking, sections were incubated at 4 °C in a humidified chamber with either a 1:200 solution of rabbit-anti-FoxP3 (ab75763, Abcam) in blocking buffer. Following overnight incubation, slides were washed in TBST, and incubated for 1 h at room temperature with a 1:200 solution of goat-anti-rabbit HRP-conjugated secondary antibody (DAKO). Sections were then washed with TBST, nuclei were counterstained with DAPI for 5 min, and the slides were coverslipped for fluorescence microscopy. For each technical replicate, the FoxP3+ nuclei were counted and summed to give a total number of FoxP3+ nuclei per biological replicate. For each biological replicate, a minimum of 2000 nuclei were counted.

### In Situ Contractile Testing

Fourteen days post-cardiotoxin injury (POD-14), in situ contractile testing protocol was implemented to evaluate the muscle’s force producing capacity^41,42^. Contractile testing was performed using an in situ testing apparatus (Model 809B, Aurora Scientific Inc, Canada), stimulator (Model 701C, Aurora Scientific Inc, Canada), and force transducer (Aurora Scientific Inc, Canada). Animals were anesthetized with 2% isoflurane. Through a small incision lateral to the knee, the peroneal nerve was isolated and exposed. The Achilles tendon was surgically cut using a scalpel prior to placing the animal supine on a 37 °C-heated platform. The foot was taped to the footplate with a surgical cloth tape, with the ankle position at 20° of plantarflexion (the position determined to result in the greatest force output)^43^. The needle electrodes were inserted beneath the skin, over the peroneal nerve. The single-twitch protocol was implemented to evaluate muscle cross-sectional area (CSA), muscle peak twitch, time to peak twitch, and half-relaxation time. Next, a force-frequency protocol was implemented by eliciting stimulations at 10, 30, 50, 80, 100, 120, 150 Hz, with a 2-minute rest between each frequency. Note that the output from the machine is torque (mN-m). Force was calculated by dividing the torque by the length of the foot plate (0.03 m). The mean CSA of the muscle was obtained using the formula: Mean CSA = (weight of TA in mg/ (length of TA in mm * density of the muscle)), where muscle density is assumed to be 1.06 g/cm^3^^44^. The specific force was then obtained by dividing the force by the mean CSA. The TA muscles were subsequently harvested and fixed in formalin for histological analysis. Peak specific force was defined as the maximum force produced during the force-frequency sweep, regardless of the frequency at which it was produced.

### MBV isolation and quantification

Small intestine was isolated from experimentally naïve mice B6 *il33*^+/+^ and B6 *il33*^−/−^ between 6–8 weeks of age. Harvested intestines were cut into ~3 cm long segments, the tunica serosa, mucosa, and muscularis externa were removed through mechanical and chemical methods as previously described^45–47^. The resulting tunica submucosa was subsequently digested overnight at 37 °C while rotating using 0.01 mg/mL solution of Liberase DL (Sigma Aldrich, St Louis, MO) in a buffer consisting of 50 mM tris (pH 8), 5 mM CaCl2, and 200 mM NaCl. Crude digest mixtures were then subjected to progressive centrifugation: 3 × 10 min at 500 × *g*, 3 × 20 min at 2500 × g, and 3 × 30 min at 10000 × g. Digests were then passed through a 0.22 μm syringe filter and ultracentrifugated at 100,000 × *g* for 2 h at 4 °C (Beckman Coulter Optima L-90K ultracentrifuge, Brea, CA). Following ultracentrifugation, supernatants were discarded and the pellet resuspended in 1 mL of particle-free PBS and further purified via size exclusion chromatography with a 10 cm column height and 1 mL fraction volume (Sepharose CL-2B beads, Sigma). Purified MBV, contained in fractions 3–5, were collected and concentrated using 100 kDa MW cut-off spin columns (Millipore, Burlington, MA). Particle concentration of each sample was determined using a NanoSight particle counter equipped with nanoparticle tracking analysis (NTA, NanoSight, Salisbury, UK).

### Exogenous MBV delivery

Two days following injury, *il33*^−/−^ mice were anesthetized using 1.5–2% inhaled isoflurane and the original incision used for CTX injection was reopened. 30 μL of IL-33^+^ MBV were gradually delivered in 1X PBS to both hindlimb TAs intramuscularly while retracting the needle from distal to proximal tendon. Pressure was applied to prevent leakage and the skin was subsequently closed with resorbable sutures and the animals were allowed to recover under supervision on a heated surface.

### Macrophage isolation, culture, and activation

Bone marrow was isolated as previously described^34^. Briefly, femurs, tibias, and fibulas were harvested from 6–8 week old mice and washed 3x in macrophage Complete Medium composed of 10% FBS (Invitrogen, Carlsbad, CA), 10% L929 supernatant, 10 mM non-essential amino acids (Gibco), 10 mM HEPES (Gibco), 2 mM L-glutamine (Gibco), 100 U/mL penicillin, 100 μg/mL streptomycin and 0.1% β-mercaptoethanol in DMEM high glucose (Gibco). Complete medium was flushed through the medullary space of harvested bones and plated at 2E6 cells/mL into 6 well plates (Corning) for 7 days until mature macrophages were obtained. Medium was supplemented 24 h after plating and changed every 48 hours thereafter. Mature macrophages were subsequently treated for 16 h with Complete Medium containing one of the following treatments: complete medium (M0 control), 20 ng/mL IFNγ + 100 ng/mL LPS (M1), 20 ng/mL IL-4 (M2), 20 ng/mL rIL-33, 1E9 IL-33^-^ MBV/mL, or 1E9 IL-33^+^ MBV/mL. If conditioned media was collected, treatments were removed and the cells washed with PBS and 500 μL of DMEM high glucose was added for 5 h. For RNA sequencing, total RNA was isolated using the RNeasy Mini Kit (Qiagen) according to the manufacturer’s instructions. RNA quantity was determined using a NanoDrop spectrophotometer (NanoDrop, Wilmington, DE).

### Quantitative polymerase chain reaction assays

RNA isolated from wildtype bone marrow-derived macrophages treated with 20 ng/mL rIL-33, 1 × 109 MBV/mL IL-33^−^, or 1 × 109 MBV/mL IL-33^+^ MBV were used for quantitative polymerase chain reaction (qPCR) as previously described^25^. Briefly, 500 ng of RNA was converted to cDNA using SuperScript III First Strand Synthesis System (Thermo Fisher) according to manufacturer’s instructions. SYBR Green (ABI) was then used to determine relative gene expression of the following transcripts: *nos2, tnfa, il1b, ccl2, il6*, and g*apdh*. Results were analyzed using the ΔΔCt method and normalized to *gapdh*. Primer sequences are listed in Supplementary Table 2. Fold change was calculated with respect to M0 controls for each replicate and averaged across replicates. Differences were assessed using two-way ANOVA with post-hoc testing.

### Primary muscle stem cell isolation and culture

Muscle stem cells (MuSCs) were isolated from the hindlimb skeletal muscle of C57BL/6 mice. Harvested muscle tissue was washed using Wash Medium consisting of HBSS with 10% horse serum and 1% penicillin/streptomycin. Hair and tendon tissue were removed and the muscle tissue was minced until it could pass through a 10 mL serological pipette. Minced muscle tissue was then subjected to serial enzymatic digestion, beginning with 750 U/mL Collagenase II in Wash Medium (Gibco, Grand Island, NY) for 60 min at 37 °C. Collagenase digested muscle was subsequently centrifuged at 900 rpm for 5 min, and the supernatant was removed. A solution of 2.4 U/mL Dispase neutral protease (Gibco) and 750 U/mL Collagenase II in Wash Medium was then added to the cell pellet. The cells were resuspended by repeated passage through a 10 mL serological pipette, and incubated for 45 min at 37 °C. The mixture was then centrifuged at 900 rpm for 5 min, the supernatant removed, and a solution of 0.1% trypsin in Wash Medium added, and incubated at 37 °C for 30 min. After completing the final enzymatic digestion, the cell mixture was centrifuged again at 900 rpm for 5 min, the supernatant removed and the cells resuspended in Wash Medium. The cell suspension was subdivided for flow sorting. The primary sort tube was incubated for 1 h with all antibodies used for selection: CD31/CD45-FITC, Sca1-APC, and ITGA7-PeCy7. A second control tube was incubated with CD31/CD45-FITC alone, and a third tube was incubated with Propidium Iodide. Compensation beads (Ultracomp eBeads, ThermoFisher Scientific, Waltham, MA) were incubated for 30 min with individual antibodies and were used to establish gating parameters. MuSCs were isolated at ≥ 95% purity by selecting for the CD31^−^/45^−^/Sca1^−^/ITGA7^+^ population^43,48^.

### MuSC differentiation assay

MuSC were seeded at 35,000 cells/mL into 4-well coverslipped chamber slides (Nunc Lab-Tek II chambered coverglass, Thermo Fisher) in MuSC Proliferation Medium containing 10% FBS (Invitrogen, Carlsbad, CA), 10% Horse Serum (Gibco), 100 U/mL penicillin, and 100 μg/mL streptomycin in DMEM high glucose (Gibco). Upon reaching ~80–90% confluency, MuSC proliferation medium was removed and replaced with low serum MuSC Differentiation Medium consisting of 10% FBS, 100 U/mL penicillin, and 100 μg/mL streptomycin in DMEM high glucose and one of the following treatments (direct treatment): 20 ng/mL recombinant IL-33 (rIL-33), 1 × 109 IL-33^*-*^ MBV/mL, or 1 × 109 IL-33^+^ MBV/mL (MBV-WT). When macrophage conditioned media were used, Proliferation Media was replaced with a 1:1 mixture of 2X Differentiation Media and one of the following treatments were added: 20 ng/mL IFNγ + 100 ng/mL LPS-treated *st2*^−/−^ macrophage conditioned media (M1 sup.), 20 ng/mL IL-4-treated *st2*^−/−^ macrophage conditioned media (M2 sup.), 20 ng/mL rIL-33-treated *st2*^−/−^ macrophage conditioned media (rIL-33 sup.), 1 × 109 IL-33^-^ MBV/mL-treated macrophage conditioned media (KO MBV sup.), or 1 × 109 IL-33^+^ MBV/mL-treated macrophage conditioned media (WT MBV sup.). Cells were allowed to differentiate for ~4 days, after which the treatment was removed, the cells washed in PBS, and fixed in 4% paraformaldehyde (PFA) for 20 min at room temperature. PFA was then removed, the cells washed 3x in PBS and blocked at room temperature in blocking buffer consisting of 0.1% Triton X, 0.1% Tween, 2% low IgG bovine serum albumin (BSA, Thermo Fisher Scientific), 4% normal goat serum (Thermo Fisher) in PBS. After 1 h, blocking buffer was removed and 1:400 mouse-anti-myosin heavy chain (MF-20c, DSHB, University of Iowa, IA) in blocking buffer was added overnight at 4 °C. The primary antibody was then removed and the cells washed 3x in PBS, and 1:500 AF546-conjugated rabbit-anti-mouse secondary antibody (Thermo Fisher) in blocking buffer was added in the dark. After 1 h, secondary antibody was removed, cells were washed 3x in PBS, and nuclei counterstained with 4′,6-diamidino-2-phenylindole (DAPI). Cells were imaged using the 10X or 20X objectives of inverted fluorescence microscope (Axio Observer Z1, Carl Zeiss, Germany). Images were quantified using CellProfiler and the fusion index was calculated as the number of nuclei contained within myotubes with ≥ 3 nuclei/tube divided by the total number of nuclei.

### Histology

Tibialis anterior muscle of wildtype mice was isolated 6–8 days post-cardiotoxin injury and MBV treatment. Tissues were fixed in formalin, paraffin-embedded, sectioned (4 µm) and stained with Masson’s trichrome using standard protocols. Using QuPath: Open source software for digital pathology image analysis^49^, blue fibrosis areas were quantified using the Train Object Classifier function. Trichrome percentage was quantified using blue fibrosis areas (mm^2^) divided by total area (mm^2^).

### Statistical analysis

Dependent variables were assessed using one-way or repeated measures ANOVA, independent samples *t*-tests. Tukey’s HSD or Sidak post-hoc testing was performed for one-way and repeated measures ANOVA, respectively. Means comparisons were performed when appropriate with an applied alpha of 0.05. Data are presented as means ± SEM unless otherwise specified. Statistical testing was performed using Graphpad Prism 8 (Graphpad, La Jolla, CA).

### Supplementary information

Supplemental Material

Reporting Checklist

## Data Availability

All relevant data supporting the findings of this study are available within the paper and from the corresponding authors upon reasonable request.
