# Supplementary material for: Matrix-bound nanovesicle-associated IL-33 supports functional recovery after skeletal muscle injury by initiating a pro-regenerative macrophage phenotypic transition
Source: NPJ Regen Med. 2024 Jan 27;9:7. doi: 10.1038/s41536-024-00346-2 (PMC10821913; doi:10.1038/s41536-024-00346-2)
Supplement: Supplementary file 1 — Supplemental Material [file 41536_2024_346_MOESM1_ESM.pdf]

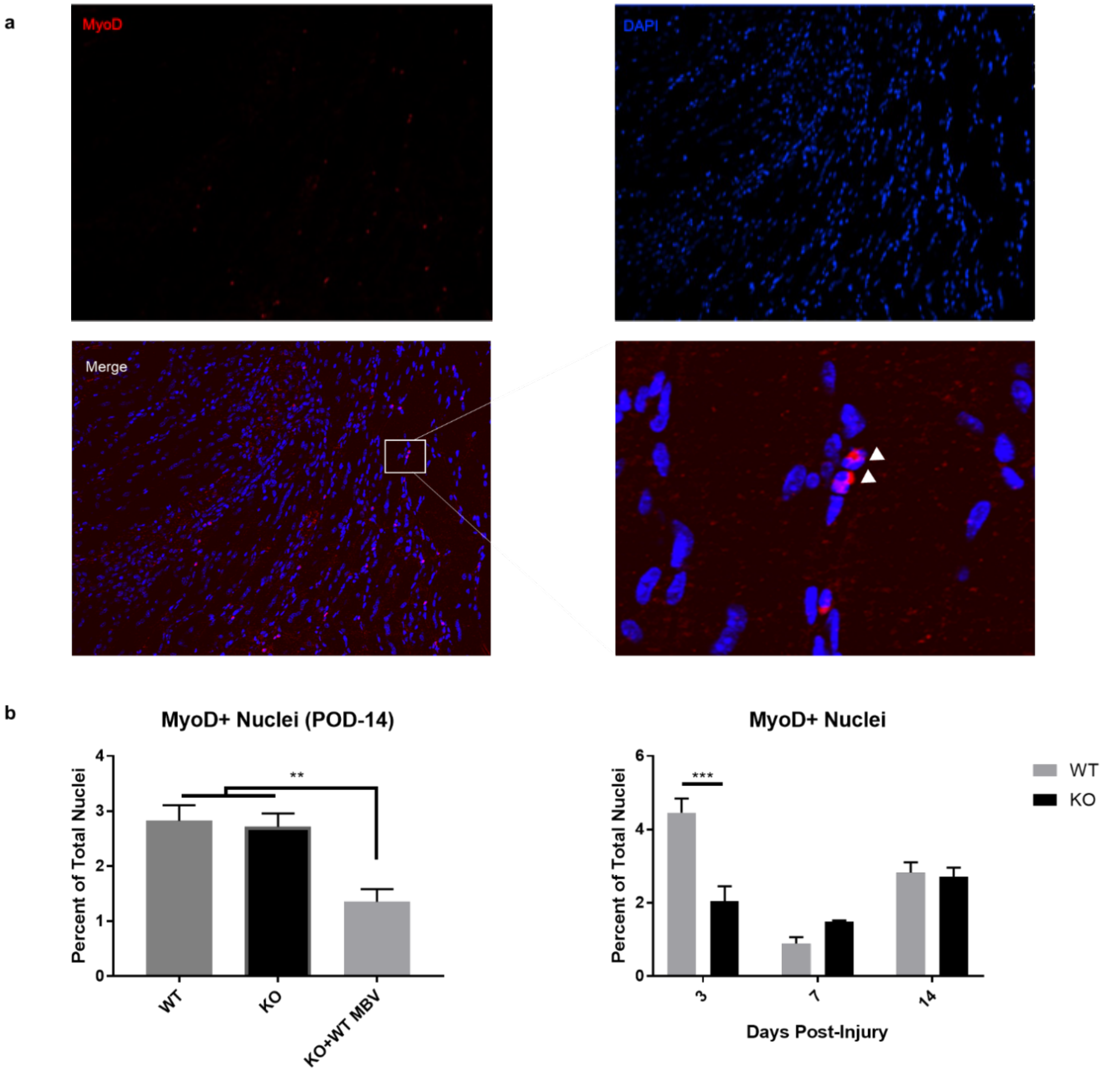

Supplemental Figure 1. ***In Situ Immunolabeling of Activated Satellite Cells.*** **(a,b)** 20X images of in situ immunolabeling of MyoD+ nuclei (a) revealed significantly fewer MyoD+ nuclei at POD3 in *il33-/-* mice than their *il33+/+* counterparts (b, right panel, $p < 0.001$), and significantly fewer MyoD+ nuclei by POD14 as a result of IL-33+ MBV treatment (b, left panel, $p < 0.01$). (N = 5 biological replicates, n $\geq$ 3 technical replicates. Data are presented as mean $\pm$ SEM, independent variables were tested for significance using one-way ANOVA (b, left panel), or multiple *t*-tests (b, right panel), ** denotes $p < 0.01$, *** denotes $p < 0.001$).

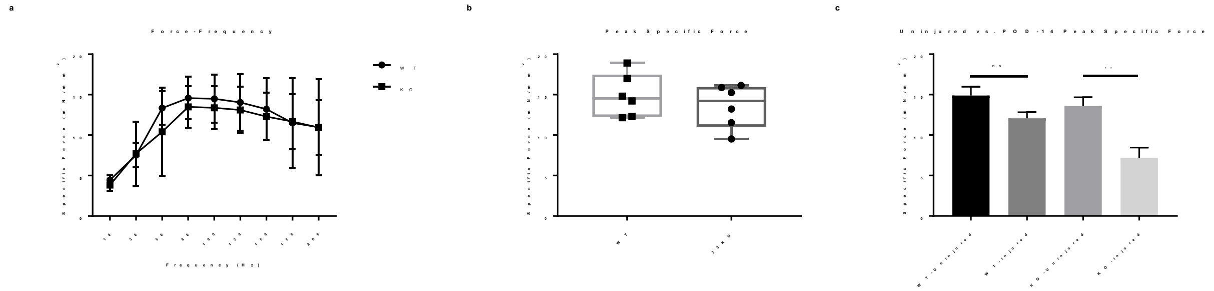

Supplemental Figure 2. ***Baseline functional characteristics of wild type and IL-33 deficient mice.*** **(a-b)** In situ contractile testing performed on uninjured and untreated *il33-/-* mice (KO) or *il33+/+* (WT) mice. No significant differences were noted between strains. **(c)** Peak specific force comparison between uninjured WT (WT-uninjured) and KO (KO-uninjured) mice vs. POD14 WT (WT-injured) and KO (KO-uninjured) mice show that WT animals are functionally restored by POD14, while KO animals are not (p < 0.01). (N = 6 biological replicates. Data are presented as mean + min/max (peak force) or mean + SEM (Force-Frequency). Independent variables were tested for significance using two-way ANOVA (Force-Frequency), independent samples *t*-test (Peak Specific Force), or one-way ANOVA followed by Tukey's post-hoc analysis (Uninjured vs. POD-14), ** denotes p < 0.01)

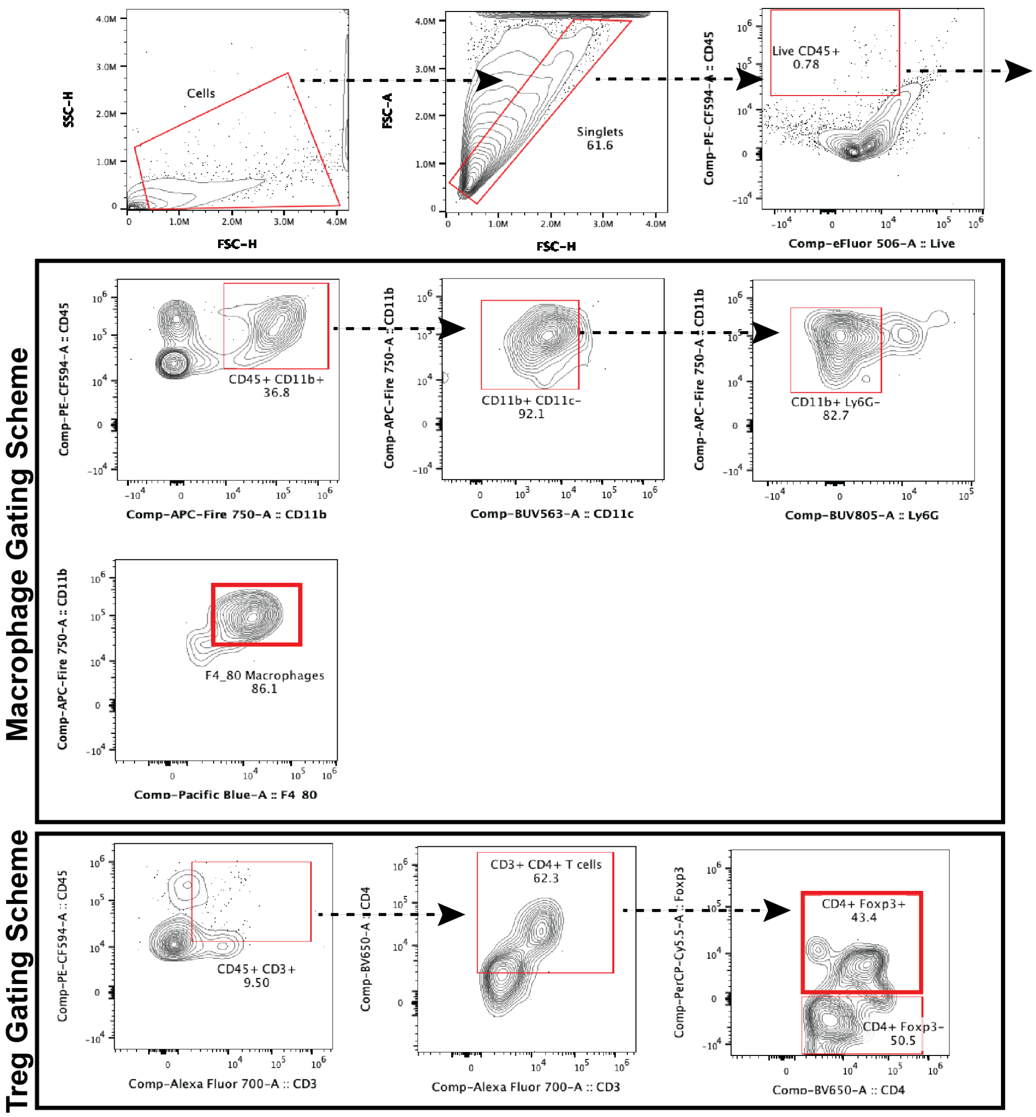

Supplemental Figure 3. ***Flow cytometry gating.*** Flow cytometry gating example to distinguish macrophage and Treg populations in mouse skeletal muscle.

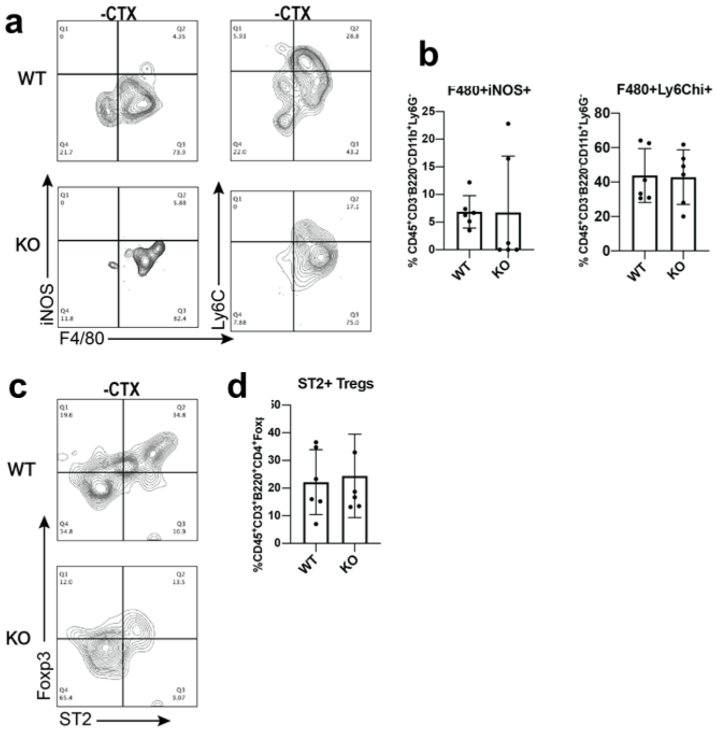

Supplemental Figure 4. ***Baseline skeletal muscle immune cell populations are non-distinct between uninjured Wild-type and Knock-out mice.*** **(a-b)** Representative dot plots and frequency for inflammatory macrophages in the CD45$^+$CD3$^-$B220$^-$CD11b$^+$Ly6G$^-$ gate (data shown as mean ± SEM). **(c-d)** Dot plots and frequency for ST2$^+$ Treg in the CD45$^+$CD3$^+$B220$^-$CD4$^+$ gate (data shown as mean ± SEM). (N = 3 biological replicates, n = 2 technical replicates. Data are shown as mean ± SEM. Variables were tested for significance using independent samples *t*-tests).

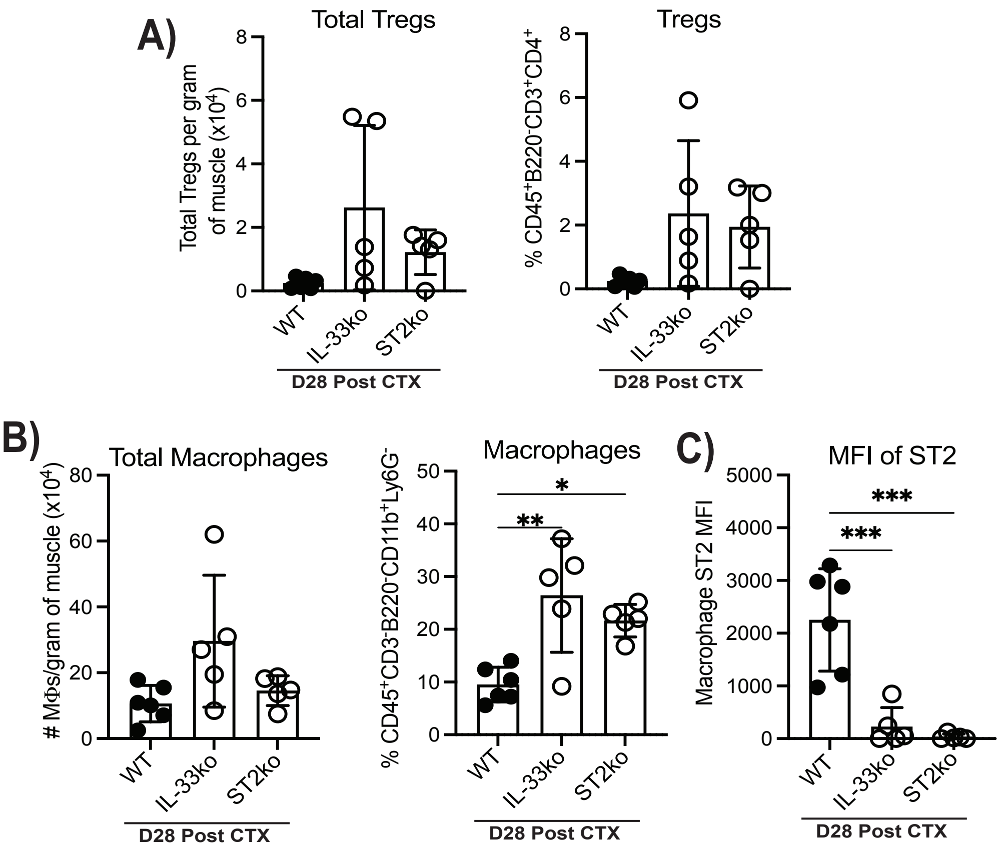

Supplemental Figure 5. Delayed immunological resolution 28 days following CTX injury due to the absence of IL-33: A) Absolute Tregs per gram of muscle (left) and Treg frequency of CD45+ live cells (right) in wildtype, IL-$33^{-/-}$, and $ST2^{-/-}$ mice 28 days following cardiotoxin injury. **B)** Absolute macrophages per gram of muscle and frequency of CD45+ live cells in wildtype, IL-$33^{-/-}$, and $ST2^{-/-}$ recipient mice 28 days following cardiotoxin injury. **C)** ST2 MFI of F4_80^{+} macrophages. N=5 or 6 per group from 2 independent experiments. Error bars are SD. ANOVA with Tukey's multiple comparisons test.

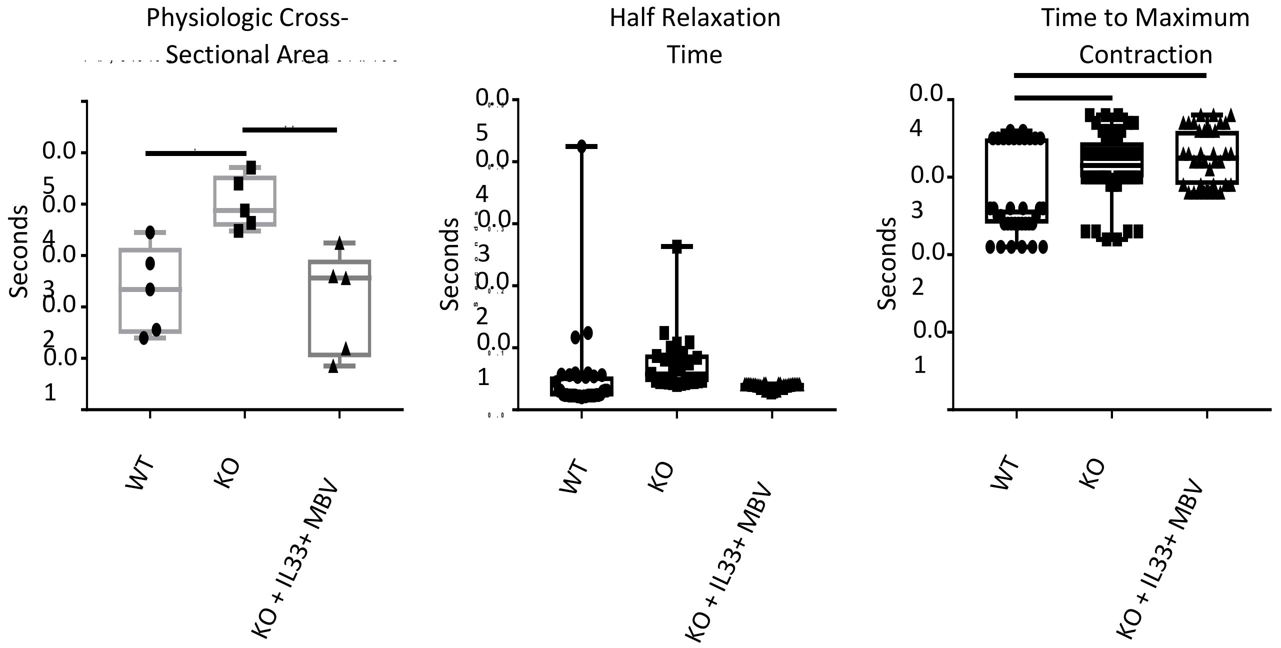

Supplemental Figure 6. ***Single stimulation contractile properties are significantly altered as a function of IL-33.*** **(a)** Physiologic cross-sectional area of untreated *il33+/+* mice (WT), untreated *il33-/-* mice (KO), or *il33*$^{-/-}$ mice to which IL-33^{+} MBV were delivered (KO + WT MBV) tibialis anterior skeletal muscle at POD14. Data revealed that IL-33^{+} MBV treatment resulted in a significantly smaller CSA compared to untreated KO skeletal muscle **(b-c)** In situ contractile measuring single stimulation parameters of WT, KO, or KO + WT MBV skeletal muscle at POD-14. Data revealed that IL-33^{+} MBV treatment resulted in no significant change in half relaxation time compared to untreated KO mice, but a significantly greater time to maximum contraction (N = 5 biological replicates, n = 6 technical replicates). Data are presented as mean $\pm$ min/max. Independent variables were tested for significance using one-way ANOVA followed by Tukey's post hoc analysis, * denotes $p < 0.05$, ** denotes $p < 0.01$, and *** denotes $p < 0.001$).

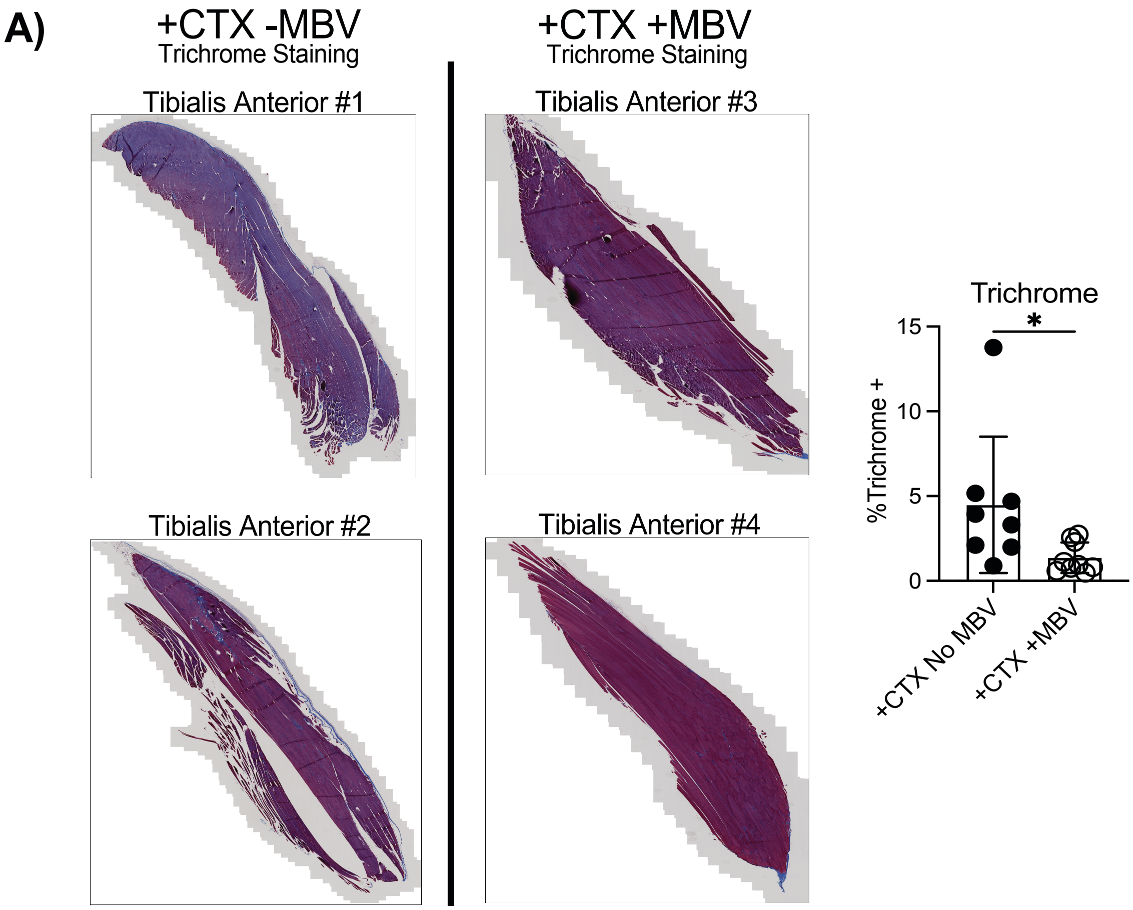

Supplemental Figure 7. ***MBV application reduces fibrotic skeletal muscle repair following sterile injury.*** **(A)** Trichrome stained sections of tibialis anterior at days 6-8 following cardiotoxin-induced muscle injury. N=5-6 per group. Error bars are SD. Student's t-test. * denotes $p < 0.05$, ** denotes $p < 0.005$, *** denotes $p < 0.001$

Supplemental Table 1. ***Raw skeletal muscle weights, CSA, and lengths as a function of genotype and treatment.***

WT Muscle Weight (g)	WT Muscle Length (mm)	WT CSA (mm^2)	KO Muscle Weight (g)	KO Muscle Length (mm)	KO CSA (mm^2)	KO + WT MBV Muscle Weight (g)	KO + WT MBV Muscle Length (mm)	KO + WT MBV CSA (mm^2)
0.035	11.88	2.779365987	0.054	12.93	3.939937837	0.048	13.8	3.281378179
0.051	12.91	3.726817006	0.061	13.7	4.200523344	0.034	12.36	2.595102888
0.042	12.49	3.1723492	0.06	12.99	4.357488344	0.034	13.22	2.42628379
0.047	12.95	3.423909084	0.056	14.12	3.741514779	0.047	12.23	3.625480183
0.035	12.24	2.697619928	0.054	13.34	3.818845294	0.043	12.3	3.298051848

Supplemental Table 2. Primer Sequences for qPCR

Target	Sequence
TNF-alpha	F: 5'-ATACACTGGCCCCGAGGCAAC-3'<br>R: 5'-CCACATCTCGGATCATGCTTTC-3'
IL-1beta	F: 5'-CTCTGTGACTCGTGGGATGATG-3'<br>R: 5'-CACTTGTTGGCTTATGTTCTGTCC-3'
IL-6	F: 5'-CCTTACTGCAGGACTTTAAGGGTTA-3'<br>R: 5'-TTTCTGGGCCATGGTTCTCT-3'
CCL2	F: 5'CATATCTGCCAAGGACATCG-3'

	R: 5’-GGTCTCTTCCATCACTTTGC-3’
Nos2	F: 5’-TGACTTCTGGCAAAATGCAG-3’<br>R: 5’-CCAAAGCGAGAGGAGTTGTC-3’
GADPH	F: 5’-GTGGTGCCAAAAGGGTCAT-3’<br>R: 5’-ATTTCTCGTGGTTCACACCCA-3’
